# miR‐155 induces ROS generation through downregulation of antioxidation‐related genes in mesenchymal stem cells

**DOI:** 10.1111/acel.12680

**Published:** 2017-10-02

**Authors:** Yuta Onodera, Takeshi Teramura, Toshiyuki Takehara, Kayoko Obora, Tatsufumi Mori, Kanji Fukuda

**Affiliations:** ^1^ Division of Cell Biology for Regenerative Medicine Institute of Advanced Clinical Medicine Kindai University Faculty of Medicine Osaka Japan; ^2^ Department of Rehabilitation Medicine Kindai University Faculty of Medicine Osaka Japan; ^3^ Kindai University Life Science Research Institute Osaka Japan

**Keywords:** aging, inflammation, mesenchymal stem cells, microRNA, stem cells, superoxide

## Abstract

Inflammation‐induced reactive oxygen species (ROS) are implicated in cellular dysfunction and an important trigger for aging‐ or disease‐related tissue degeneration. Inflammation‐induced ROS in stem cells lead to deterioration of their properties, altering tissue renewal or regeneration. Pathological ROS generation can be induced by multiple steps, and dysfunction of antioxidant systems is a major cause. The identification of the central molecule mediating the above‐mentioned processes would pave the way for the development of novel therapeutics for aging, aging‐related diseases, or stem cell therapies. In recent years, microRNAs (miRNAs) have been shown to play important roles in many biological reactions, including inflammation and stem cell functions. In inflammatory conditions, certain miRNAs are highly expressed and mediate some cytotoxic actions. Here, we focused on miR‐155, which is one of the most prominent miRNAs in inflammation and hypothesized that miR‐155 participates to inflammation‐induced ROS generation in stem cells. We observed mesenchymal stem cells (MSCs) from 1.5‐year‐old aged mice and determined that antioxidants, Nfe2l2, Sod1, and Hmox1, were suppressed, while miR‐155‐5p was highly expressed. Subsequent *in vitro* studies demonstrated that miR‐155‐5p induces ROS generation by suppression of the antioxidant genes by targeting the common transcription factor C/ebpβ. Moreover, this mechanism occurred during the cell transplantation process, in which ROS generation is triggering loss of transplanted stem cells. Finally, attenuation of antioxidants and ROS accumulation were partially prevented in miR‐155 knockout MSCs. In conclusion, our study suggests that miR‐155 is an important mediator connecting aging, inflammation, and ROS generation in stem cells.

## Introduction

Chronic inflammation is associated with normal and pathological aging (Chung *et al*., 2009). Multiple interrelated and complex mechanisms contribute to inflammation and aging, and levels of inflammatory mediators typically increase with age even in the absence of acute infection or other physiologic stress (Singh & Newman, 2011). In the context of inflammation and aging, reactive oxygen species (ROS) are generally the major molecules contributing to the acceleration of inflammation‐related tissue degeneration and aging. Evidence demonstrates that aging, oxidative stress, and inflammation are interdependent mechanisms (Sarkar & Fisher, 2006; Chung *et al*., 2009).

ROS is induced by pro‐inflammatory stimulators such as TNFα (Schulze‐Osthoff *et al*., 1993) and IL1β (Hwang *et al*., 2004) and participates to inflammation‐related tissue degeneration. In normal conditions, ROS generation is essential for cell signaling and several important physiological responses (Zhang *et al*., 2016). However, ROS is highly reactive and toxic. It is finely controlled by various antioxidants such as superoxide dismutase (SOD), hemeoxygenase‐1 (HMOX1), and nuclear factor erythroid 2‐related factor 2 (NFE2L2), which is known as a master regulator of these genes (Fujita *et al*., 2012; Cheng *et al*., 2013). In aged tissues/organs and in several diseases such as neurodegeneration and cancer, these are associated with highly inflammatory conditions and excessive ROS accumulation is observed (Jomova & Valko, 2011). High amount of ROS generation or deregulation of antioxidant systems can lead to cellular dysfunction, abnormality, and cell death and result in further inflammation and organ/tissue aging (Mittal *et al*., 2014). When excessive ROS generation arises in stem cells, it directly leads to deterioration of tissue turnover and regeneration. Excess ROS can impair self‐renewal, differentiation capacity, and proliferation of stem cells (Oh *et al*., 2014; Denu & Hematti, 2016). Therefore, accumulation of ROS and deterioration of stem cell abilities are considered central mechanisms in the aging process.

In this study, we focused on mesenchymal stem cells (MSCs). MSCs are multipotent tissue stem cells for skeletal tissues characterized by surface markers such as CD105, CD44, and PDGFRα (Morikawa *et al*., 2009). In response to growth factors, they express chondrogenic/osteogenic transcription factors and differentiate into chondrocytes or osteoblasts. Thus, reduction in the number of MSCs in the bone marrow (BM) or imbalance of MSC differentiation leads to senile osteoporosis caused by declined bone formation (Rachner *et al*., 2011). Aging/inflammation/over‐accumulation of ROS in MSCs is also detrimental to some nonskeletal tissues, in particular for the hematopoietic system, where MSCs are essential as a niche component (Morrison & Scadden, 2014). Furthermore, MSCs are now studied for their regenerative and immunomodulatory properties, as they home to injured tissues and contribute to tissue regeneration (Squillaro *et al*., 2016). In this context, loss of transplanted MSCs at the grafted site seems to hinder the results of MSC transplantation. Nonspecific inflammation at the ischemic site of injury and ROS generation have been hypothesized as major factors for the loss of MSCs at the grafted site (Pittenger & Martin, 2004). Therefore, the identification of the molecular mechanisms connecting inflammation and ROS generation is greatly needed to understand stem cell aging and to improve stem cell therapy. The identification of the central player mediating this process is essential.

Recently, microRNAs (miRNAs), noncoding RNA of 19–25 nucleotides, have been reported to play critical roles in many cellular processes such as proliferation, differentiation, and cell viability (Bartel, 2009). In inflammation‐related reactions, some miRNAs are upregulated and contribute to degenerative reactions (Marques‐Rocha *et al*., 2015). However, whether miRNA can mediate inflammation and ROS generation remains unclear. Here, we hypothesized that miRNAs induced by pro‐inflammatory stimulation are involved in the regulation of ROS generation. We demonstrated that miR‐155 is overexpressed in the BM of aged mice and is involved in ROS generation by suppressing the antioxidant systems controlling cellular ROS generation in mouse and human MSCs.

## Results

### Antioxidant genes and miR‐155‐5p expression and ROS levels in young and aged mouse bone marrow (BM) tissues

**Figure 1:**
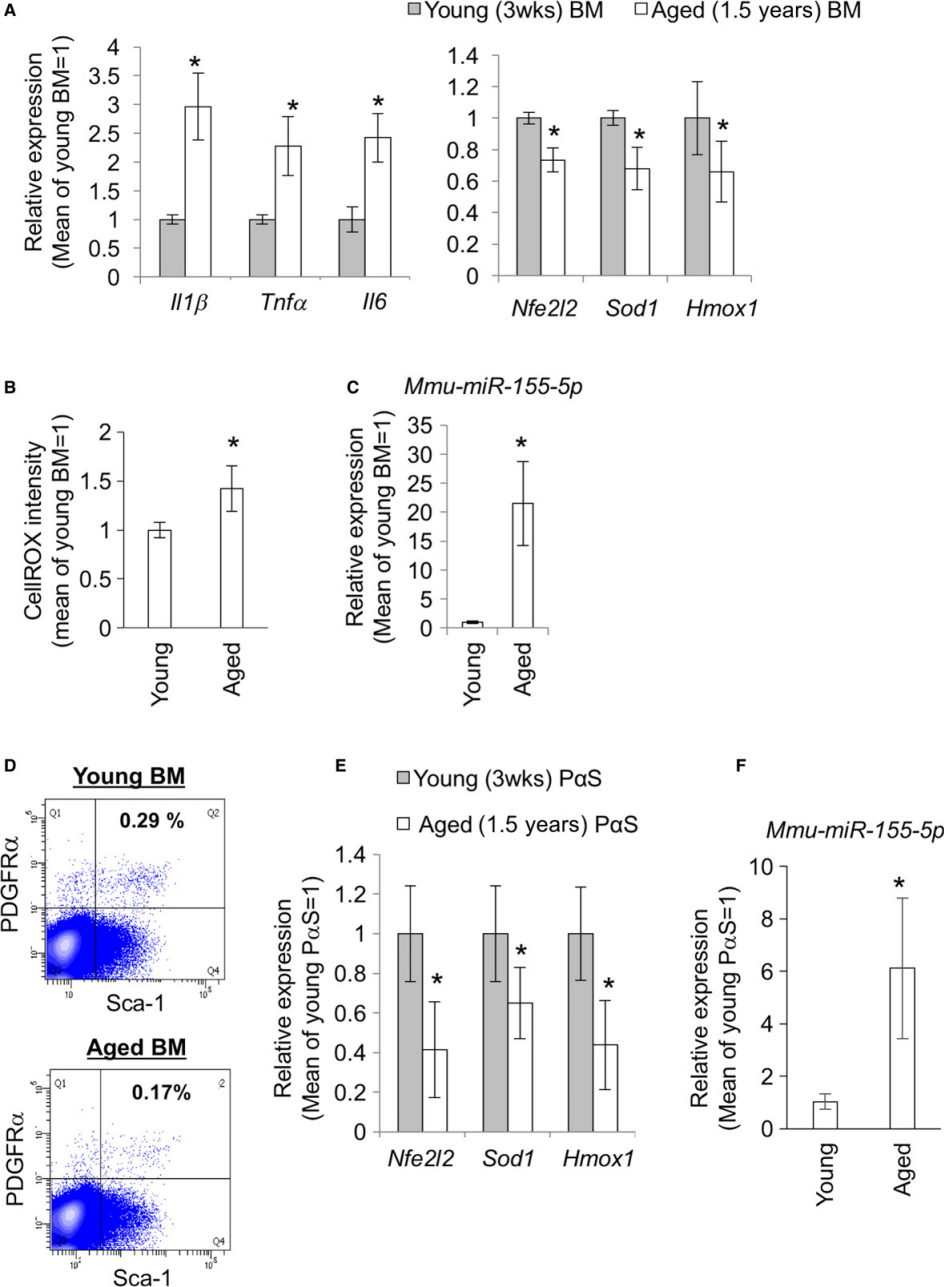
Expression of inflammatory cytokines, antioxidation‐related genes, ROS and miR‐155 in BM tissues and mesenchymal stem cells from young and aged mice. (A) Relative expression levels of inflammatory cytokines, *Il1*β, *Tnf*α*,* and *Il6,* and antioxidant genes, *Nfe2l2*,* Sod1,* and *Hmox1*. Six times of independent experiments repeated (*n *= 6). The y‐axis shows the relative expression levels to mean values for each gene in young (3 weeks old) mice. (B) ROS levels in BM tissues of young (3 weeks old) and aged (1.5 years old) mice. (C) Relative expression levels of *mmu‐miR‐155‐5p* in BMs from young (3 weeks old) and aged (1.5 years old) mice (*n *= 6). (D) PDGFRα/ Sca1 double‐positive (PαS) MSC fractions for the gene expression analysis. Data of one representative experiment are shown. (E) relative expression levels of antioxidant genes, *Nfe2l2*,* Sod1*, and *Hmox1,* in the PαS MSCs from young and aged mice (*n *= 6). (F) Relative expression levels of *mmu‐miR‐155‐5p* in the PαS MSCs from young and aged mice (*n *= 6). The asterisks represent significant differences (*P* < 0.05) compared with young BM samples.

Increased expression of inflammatory cytokines and ROS accumulation have been observed in aged tissues. In this study, we determined whether the expression of inflammatory cytokines and ROS were upregulated in aged BM tissues. The expression of *Il1*β, *Tnf*α*,* and *Il6* were upregulated in aged BM tissues. In contrast, gene expression of antioxidant‐related proteins, *Nfe2l2*,* Sod1,* and *Hmox1,* was suppressed (Figs 1A and S1, Supporting information). ROS were upregulated about 1.5 times compared with that in the BM of young mice (Fig. 1B). Additionally, the expression level of *mmu‐miR‐155‐5p* was 20‐times higher than that in the BM of young mice (Fig. 1C).

### Antioxidant genes are suppressed, while miR‐155‐5p is upregulated in PDGFRα/Sca1 double‐positive (PαS) MSCs

To assess age‐related expression changes in the expression of antioxidant genes and miR‐155‐5p, we isolated MSCs from young and aged mouse BM tissues. PDGFRα/Sca1 double‐positive (PαS), which are selective markers of mouse mesenchymal stem cells (MSCs) (Zhu *et al*., 2010), fractions were collected at 0.32% (mean of 7 trials) from young BMs and 0.17% (mean of 14 trials) from aged BMs after collagenase treatment and FACS sorting (Fig. 1D). In the PαS MSCs from aged mice, gene expression of *Nfe2l2*,* Sod1,* and *Hmox1* was attenuated (Fig. 1E), while *miR‐155‐5p* expression was upregulated (Fig. 1F).

### Exposure to inflammatory cytokines generates ROS in mouse MSCs

**Figure 2:**
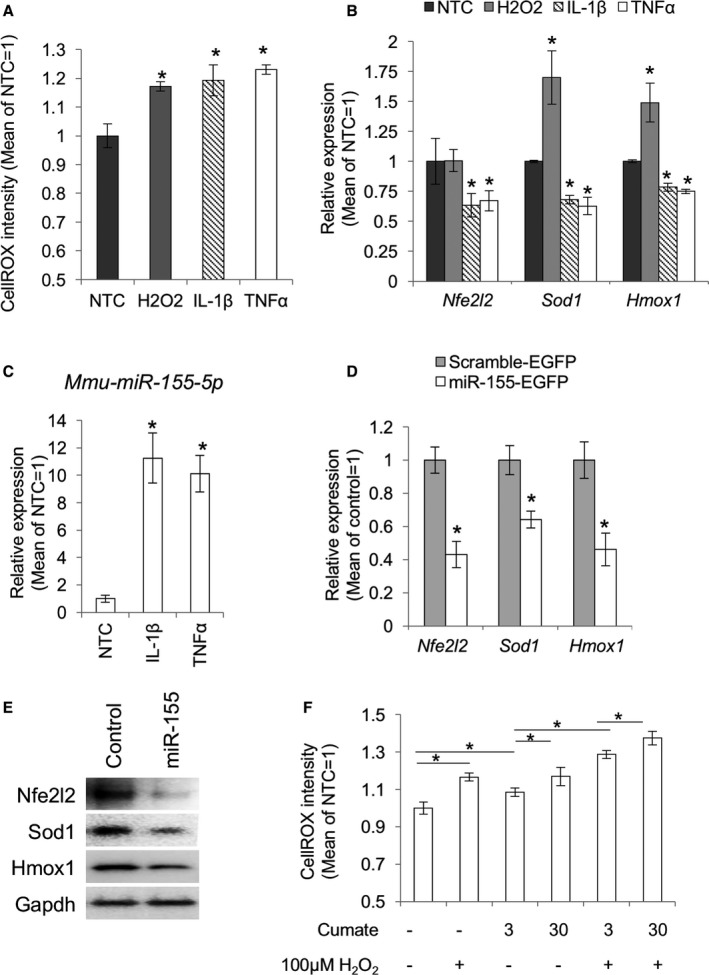
miR‐155 induced ROS accumulation through suppression of antioxidation‐related genes in the cultured mouse MSCs. (A) Increased cellular ROS in the inflammatory cytokine‐stimulated MSCs. Nontreatment control (NTC) means cells treated with PBS followed by CellROX‐dye. (B) qPCR for the antioxidant genes, *Nfe2l2*,* Sod1,* and *Hmox1* (*n *= 3). Cells were analyzed at 12 hours after administration of H_2_O_2_, 1 ng mL^−1^ IL1β, or 10 ng mL^−1^ TNF **α**. Asterisks represent significant differences compared with the nontreated control (NTC) at *P *< 0.05. (C) IL1β and TNF **α**‐induced *mmu‐miR‐155‐5p* expression in the cultured mouse MSCs. U6 small nuclear RNA (snRNA) was used as an internal control. The asterisks represent a significant difference (*P *< 0.05) compared with the NTC (*n *= 3). (D) qPCR for *Nfe2l2*,* Sod1,* and *Hmox1* in MSCs transfected with the EGFP‐mmu‐miR‐155 expression plasmid (*n *= 3). Asterisks represent significant differences between control (*P *< 0.05). (E) Western blots for *Nfe2l2*, Sod1, and Hmox1 in the MSCs transfected with the EGFP‐*mmu‐miR‐155‐*expression plasmid. (F) ROS levels in MSCs transfected with the cumate induction system for miR‐155 (pPBQM‐*mmu‐miR155*) followed by treatment with cumate at 3 or 30 μg mL^−1^. H_2_O_2_ treatment was set as a positive control for ROS accumulation (*n *= 3). Asterisks represent significant differences (*P *< 0.05) between the groups connected by bars.

ROS generation and *miR‐155‐5p* upregulation can be induced by inflammatory cytokines in multiple cell types. However, this is not evidenced well in MSCs. Therefore, we assessed the effect of IL1β and TNFα on ROS generation in MSCs using CellROX‐dye. The positive control comprising MSCs treated with H_2_O_2_ showed an increase in the CellROX fluorescence, and both IL1β and TNFα upregulated cellular ROS (Fig. 2A). We then examined whether ROS generation by inflammatory cytokines in MSCs resulted from the downregulation of redox genes by observing the expression of the antioxidant genes, *Nfe2l2*,* Sod1,* and *Hmox1*. TNFα and IL1β addition clearly affected *Nfe2l2*,* Sod1,* and *Hmox1* gene expression (Fig. 2B).

### miRNA‐155 inhibits the expression of redox genes, *Nfe2l2, Sod1,* and *Hmox1* in mouse MSCs

We then measured the expression level of *miR‐155‐5p* after stimulation with IL1β and TNFα and observed that expression of *mmu‐mir‐155‐5p* was strongly upregulated (Fig. 2C). Additionally, overexpression of *mmu‐mir‐155* in MSCs resulted in a decrease in the mRNA and protein expression of *Nfe2l2*, Sod1, and Hmox1 (Fig. 2D,E). We then examined whether the downregulation of redox genes led to ROS generation in MSCs using an inducible expression system of *mmu‐mir‐155*. In this system, *mmu‐mir‐155* expression can be induced in a dose‐dependent manner by addition of cumate (Fig. S2). Induction of *mmu‐mir‐155* by cumate led to increase in CellROX fluorescence, indicating that miR‐155 expression is responsible for the accumulation of cellular ROS (Fig. 2F).

### miR‐155 attenuates redox gene expression by suppressing C/ebpβ

**Figure 3:**
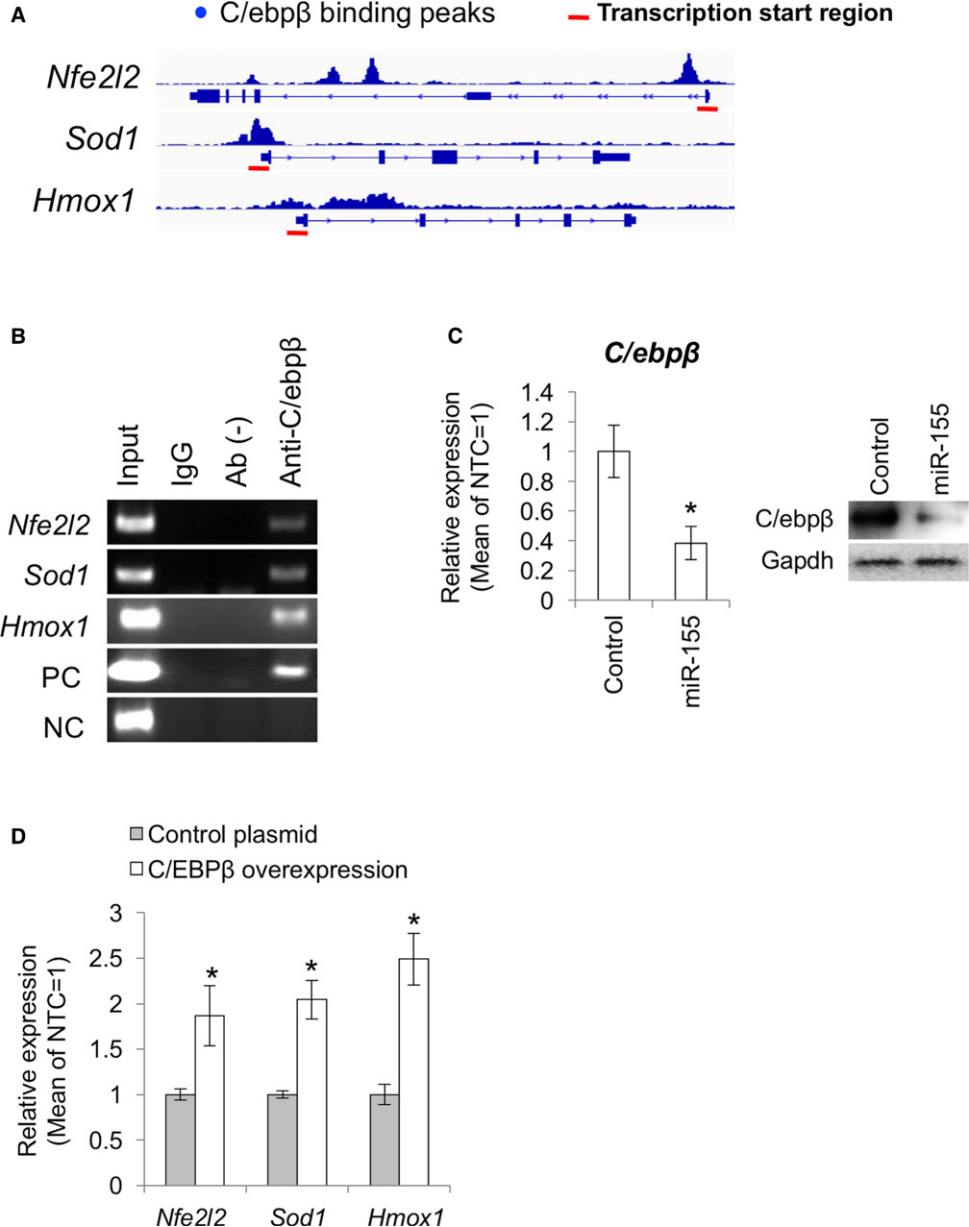
*C/ebp*β is involved in the regulation of *Nfe2l2, Sod1,* and *Hmox1* expression in the MSCs. (A) Putative C/ebpβ binding regions on the *Nfe2l2, Sod1,* and *Hmox1* genes. Red bars show transcription start regions. (B) ChIP‐PCR analysis with the C/ebpβ antibody for the *Nfe2l2, Sod1,* and *Hmox1* promoters. (C) C/ebpβ expression in the MSCs transfected with *mmu‐miR‐155* overexpression plasmid (*n *= 3). Control means the MSCs transfected with the control plasmid containing a scrambled sequence. Asterisk represents significant differences (*P *< 0.05) compared with control. (D) qPCR for *Nfe2l2, Sod1,* and *Hmox1* in the MSCs transfected with the *C/ebp* ***β*** overexpression plasmid. Asterisks represent significant differences (*P *< 0.05) compared with control.

We hypothesized that molecular relationships for the regulation of important cellular homeostasis or disease development should be conserved in both human and mice. Thus, we searched target genes listed up as common targets of *mmu‐mir‐155‐5p* and *hsa‐mir‐155‐5p* in the TargetScan and DIANA‐microT programs. As prediction scores for *Nfe2l2*,* Sod1,* and *Hmox1* were not significant using our criteria, we hypothesized that the attenuation of the expression of *Nfe2l2*,* Sod1,* and *Hmox1* by miR‐155 was mediated by regulation of a common transcription factor. From the results of *in silico* ChIP analysis and previous studies, we hypothesized that C/ebpβ mediates the effect of miR‐155 on the expression of antioxidant genes. By analyzing ChIP‐seq data from previous studies (Meyer *et al*., 2016), we determined that C/ebpβ can bind to the promoter region of *Nfe2l2*,* Sod1,* and *Hmox1* (Fig. 3A). To confirm that C/ebpβ is involved in the regulation of the antioxidant genes, we performed ChIP‐PCR analysis with anti‐C/ebpβ antibody for promoter regions of *Nfe2l2*,* Sod1,* and *Hmox1*. ChIP‐PCR indicated the binding of C/ebpβ on the putative binding regions of *Nfe2l2*,* Sod1,* and *Hmox1* promoters (Fig. 3B). We then assessed C/ebpβ expression levels in *mmu‐miR‐155*‐overexpressing MSCs by qPCR and Western blot. In the MSCs transfected with the *mmu‐mir‐155* coding plasmid, C/ebpβ expression was significantly inhibited (Fig. 3C). We then overexpressed C/ebpβ cDNA in MSCs to determine that the binding was functional and confirmed that C/ebpβ overexpression resulted in an increase in the expression of *Nfe2l2*,* Sod1,* and *Hmox1* (Fig. 3D). These results indicated that C/ebpβ is an important transcription factor involved in the upregulation of these genes.

### miR‐155‐5p and ROS are upregulated in transplanted MSCs

**Figure 4:**
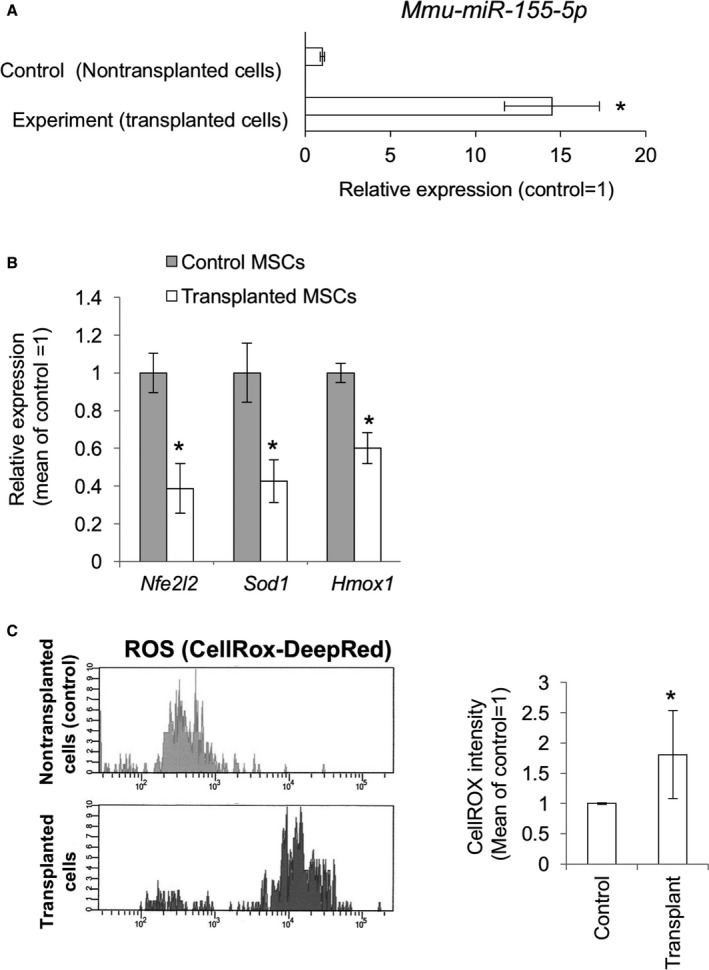
Gene expressions and ROS generations in the MSCs transplanted to the posterior biceps femoral (PBF) muscles of mice. (A) *mmu‐miR‐155‐5p* expression level in the transplanted MSCs after 48 hours of operation (*n *= 6). The asterisk represents a significant difference (*P *< 0.05) compared with the control. (B) Relative gene expression levels of *Nfe2l2, Sod1,* and *Hmox1* in the control and transplanted cells (*n *= 6). (C) FACS analysis for ROS accumulation with the CellROX‐DeepRed dye. The upper panel represents fluorescence intensity in the control cells treated with the CellROX. The lower panel represents fluorescence intensity in the transplanted cells treated with the CellROX. Cells were discriminated from endogenous cells by GFP fluorescence. (D) Mean scores of the CellROX fluorescence intensity in the control and transplanted cells (*n *= 6). Asterisks represent significant differences (*P *< 0.05) compared with the control.

Excessive inflammation and ROS generation are common problems that need to be resolved in tissue homeostasis and regeneration and for stem cell therapies during cell transplantation. We then hypothesized that *miR‐155‐5p* was upregulated in transplanted cells and participated in the deregulation of redox signaling. To this end, we prepared MSCs from EGFP transgenic mice, transplanted them into B6 mice, and analyzed gene expression and ROS accumulation after 48 h of transplantation. Consistent with our hypothesis, *mmu‐miR‐155* expression was significantly upregulated in the transplanted cells (Fig. 4A). Furthermore, gene expression of *Nfe2l2*,* Sod1,* and *Hmox1* was attenuated in the transplanted cells (Fig. 4B). Consistently, CellROX fluorescence was significantly increased compared with that in control cells (Fig. 4C,D).

### Suppression of *Nfe2l2*,* Sod1,* and *Hmox1* expression and ROS generation are mitigated in miR‐155 knockout cells

**Figure 5:**
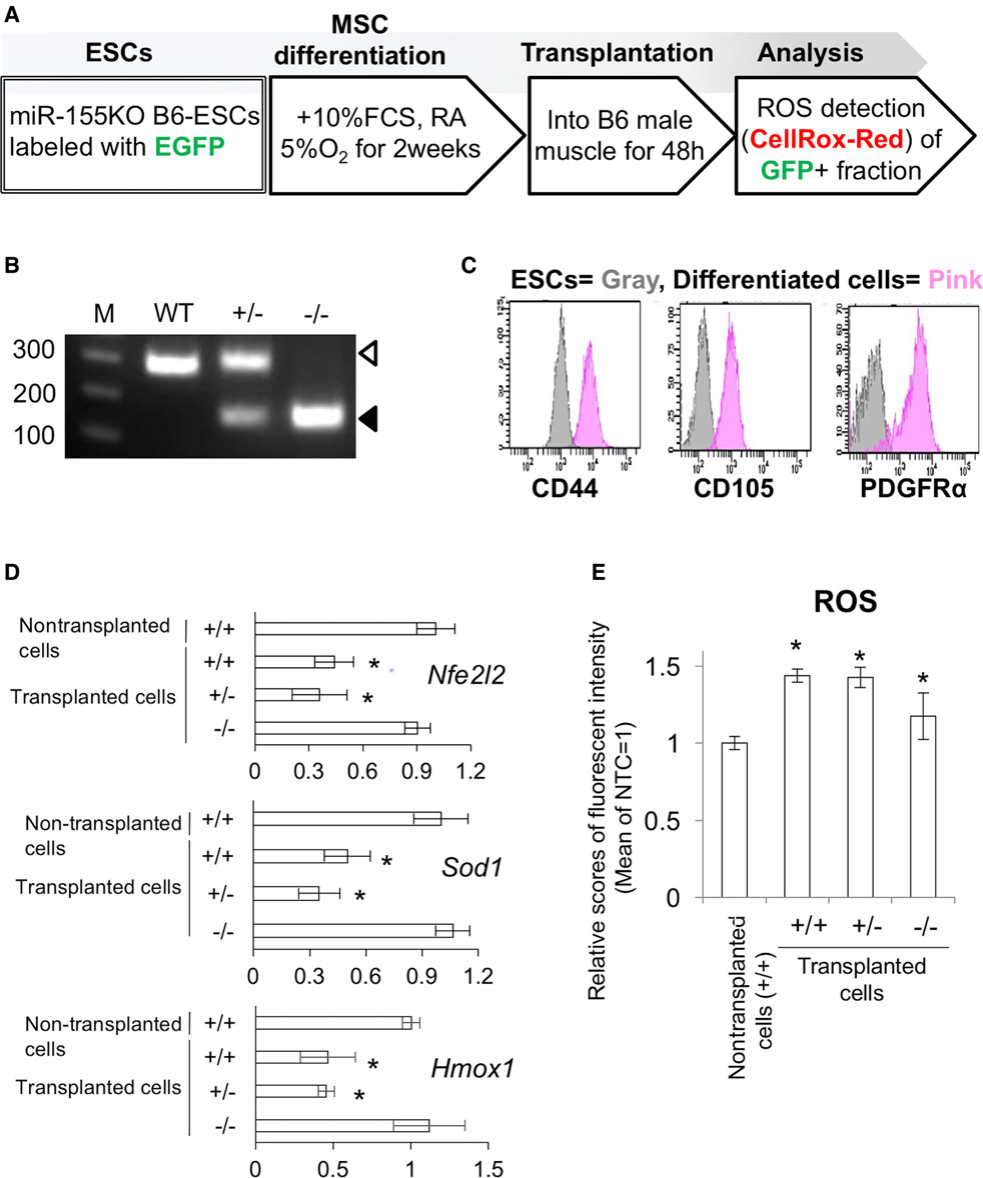
miR‐155 knockout cells transplantation experiment. (A) Scheme representation of *in vitro* MSC induction, cell transplantation, and ROS detection experiments. (B) Genotyping for the miR‐155 genomic locus in the CRISPR‐treated ESCs. The white arrowhead represents the 296‐bp band for the wild‐type (WT), and the black arrowhead represents 153‐bp band for the miR‐155 deleted mutant. (C) FACS analysis of the *in vitro* induced MSCs from the ESCs. (D) qPCR for *Nfe2l2, Sod1,* and *Hmox1* in the WT, hetero, and knockout cells after transplantation (*n *= 6). (E) ROS detection in the WT, hetero, and knockout cells after transplantation (*n *= 6). Asterisks represent significant differences (*P* < 0.05) compared with control.

To demonstrate the involvement of miR‐155‐5p in the suppression of antioxidant genes and ROS accumulation, we performed CellROX assays using miR‐155 knockout cells (Fig. 5A). To this end, we produced miR‐155 knockout mouse ESCs by CRISPR‐Cas9 with two guide RNAs. In the knockout cells, the target region containing *miR‐155* sequence was deleted (Figs 5B and S3). The knockout ESCs were labeled with piggyback‐GFP plasmid, and MSC differentiation was induced *in vitro* as previously reported (Teramura *et al*., 2010). Induced MSCs showed typical spindle‐shaped fibroblast‐like morphology and expressed MSC markers, CD105, CD44, and CD140α (PDGFRα) (Fig. 5C). Moreover, in the ESC‐derived MSCs, *Nfe2l2*,* Sod1,* and *Hmox1* expression and subsequent ROS generation were suppressed by transplantation. However, this effect was blocked in MSCs derived from miR‐155 knockout ESCs (Fig. 5D). ROS level was also inhibited in the knockout cells compared with that in wild‐type and heterozygous knockout cells, although the fluorescence level was higher than that in the nontransplanted control (Fig. 5E).

### The molecular pathway involving miR‐155, C/EBPβ, and redox‐related genes, *NFE2L2, SOD1*, and *HMOX1,* is conserved in human MSCs

**Figure 6:**
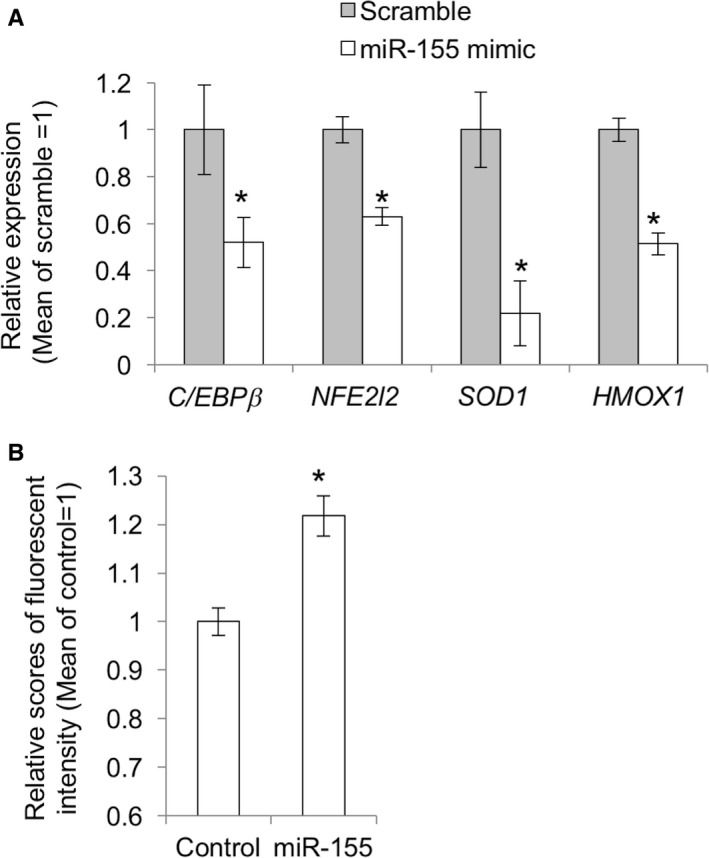
miR‐155‐induced reduction of the expression of antioxidant genes and ROS generation in human MSCs. (A) qPCR for *Nfe2l2, Sod1,* and *Hmox1* in MSCs treated with mimic RNA for *hsa‐miR‐155‐5p* for 48 h (*n *= 3). The asterisk represents a significant difference (*P *< 0.05) compared with the control that was treated with scrambled RNA. (B) ROS detection in the *hsa‐miR‐155‐5p* mimic treated human MSCs by CellROX.

Finally, we examined whether the molecular pathway described above was conserved in humans using human BM‐MSCs. Treatment with a *hsa‐miR‐155‐5p* mimic resulted in the suppression of *C/EBP*β*, NFE2L2, SOD1,* and *HMOX1* expression (Fig. 6A). Consistently, ROS was increased by treatment with *hsa‐miR‐155‐5p* mimic (Fig. 6B). These results suggest that the mechanisms underlying miR‐155‐mediated ROS generation are common between mouse and human cells.

## Discussion

Tissue repair and regeneration are of prime importance for the maintenance of tissue homeostasis. They depend on maintaining the functional capacity in tissue‐specific stem cells, but this functionality decreases with aging, partially because of ROS accumulation associated with inflammation (Oh *et al*., 2014).

Here, we demonstrated that the expression levels of inflammatory cytokines, IL1β and TNFα, are upregulated in the BM tissues of aged mice and, consistently, high amounts of ROS are observed. Importantly, miR‐155‐5p expression level was 15‐times higher in aged BM tissues than that in young mice. Furthermore, miR‐155‐5p upregulation and decreased expression of antioxidant genes were also detected in PDGFRα/Sca1 double‐positive MSCs from the aged BM tissues. These results are consistent with previous observations in different tissues showing that miR‐155 is upregulated by aging (Sredni *et al*., 2011; Park *et al*., 2013). It is well known that miR‐155 is positively regulated by pro‐inflammatory reactions (Arts *et al*., 2015; Qayum *et al*., 2016); however, whether miR‐155 is involved in the molecular network connecting inflammation and ROS generation remains unknown. We performed an *in vitro* study using cultured MSCs and determined that IL1β and TNFα induced the downregulation of antioxidant genes and increased ROS levels. The inflammation‐induced suppression of antioxidants observed in this study is consistent with previous studies (Agharazii *et al*., 2015). Additionally, it has been shown that attenuation of antioxidant genes can lead to an increase in cellular ROS and degeneration of various tissues (Langer *et al*., 1996; Harijith *et al*., 2014). Thus, we concluded that IL1β or TNFα stimulation induced ROS generation partially by attenuating antioxidant gene expression.

Recent studies indicated the important roles of miRNAs in ROS generation through targeting genes related to antioxidant responses such as Sirtuins (Zhu *et al*., 2011; Lang *et al*., 2016). However, the direct relation between miR‐155 and antioxidant signaling pathways remains unclear. To confirm that the attenuation of *Nfe2l2*, Sod1, and Hmox1 expression was mediated by *miR‐155‐5p*, we introduced an EGFP‐mmu‐miR‐155 expression plasmid and a cumate‐inducible *mmu‐miR‐155* expression plasmid into the mouse MSCs. Overexpressed miR‐155 suppressed antioxidant genes and triggered ROS generation. As no significant relationships between miR‐155 and *Nfe2l2*, Sod1, or Hmox1 were identified using our criteria; that is, either *mmu‐miR‐155‐5p* or *hsa‐miR‐155‐5p* did not show significant probability of binding to these genes, we excluded the possibility that *Nfe2l2*, Sod1*,* and Hmox1 are directly targeted by miR‐155‐5p. We performed an *in silico* analysis using miRNA target prediction programs and GEO ChIP‐seq datasets and hypothesized that miR‐155 targets a master transcriptional factor regulating *Nfe2l2*, Sod1*,* and Hmox1. Among the transcription factors that regulate antioxidant expression, we focused on C/ebpβ. C/ebpβ is one of the major transcription factors targeted by miR‐155 and could be a common regulator of many homeostatic genes involved in antioxidation including *Sod1* (Seo *et al*., 1996) and *Hmox1* (Mrad *et al*., 2012). It has been reported that the promoter of *Nfe2l2* lacks the CCAAT box (Chan *et al*., 1996), and no distinct evidence suggests its regulation by C/EBPβ. However, a clear peak was detected in our ChIP‐seq analysis, suggesting C/EBPβ binding on a site close to the transcription start site of *Nfe2l2,* and the binding was verified by ChIP‐qPCR. C/EBPβ overexpression increased the expression of *Nfe2l2, Sod1,* and *Hmox1*. These results suggest that C/ebpβ positively regulates gene expression of *Nfe2l2*,* Sod1,* and *Hmox1* through direct binding to their promoter regions. Previous evidence shows that *Nfe2l2* is a key transcription factor that regulates antioxidant systems, including control of Sod1 and Hmox1 (Cheng *et al*., 2013). Therefore, it is possible that oxidized stress‐specific gene transcription is regulated by *Nfe2l2,* while C/EBPβ may regulate ubiquitous transcriptional activity.

Based on the above observations, we conclude that miR‐155, which is upregulated in aging or aging‐associated inflammation in BM tissues, suppresses C/ebpβ and its downstream target genes *Nfe2l2, Sod1,* and *Hmox1*, thereby inducing ROS generation in MSCs. Thus, miR‐155 might be an important player connecting inflammation and ROS generation.

In another model of inflammation‐induced ROS generation in MSCs, we hypothesized that miR‐155 expression involves ROS generation during MSC transplantation. Currently, MSC transplantation is utilized for the treatment of various diseases, and many positive clinical effects have been reported. However, the engraftment rate of stem cell transplantation is considered a limitation of the therapy (Liu *et al*., 2010; Semont *et al*., 2010). Some research groups found that transplanted cells are suddenly exposed to a large amount of inflammatory cytokines during the cell transplantation process, and the transplanted cells generate excessive ROS, triggering cell death (Wei *et al*., 2010). High level of ROS can activate some stress kinases such as apoptosis signal‐regulating kinase‐1 (ASK1), p38, and stress‐activated protein kinase (SAPK)/Jun‐N‐terminal kinase (JNK) pathways (Matsuzawa *et al*., 2005). These pathways can lead to accumulation of cellular stresses, inappropriate differentiation, and reduction of cell proliferation. Therefore, managing ROS generation during cell transplantation would be an effective way to improve the rate of stem cell transplantation and may be the key to control stem cell aging caused by inflammation‐associated ROS accumulation. Consistent with our hypothesis, *miR‐155‐5p* expression was upregulated more than 10 times in the transplanted cells, and attenuation of *Nfe2l2, Sod1,* and *Hmox1* expression in the transplanted cells was also observed at 48 h after operation. Furthermore, ROS levels were significantly upregulated in the transplanted cells. To confirm that ROS generation was induced by *miR‐155‐5p* expression, we produced miR‐155 knockout MSCs from ESCs, in which miR‐155 coding region was deleted by CRISPR‐Cas9 technology, and detected intracellular ROS after cell transplantation. At 48 h after transplantation, *Nfe2l2, Sod1* and *Hmox1* expression decreased in wild‐type and heterozygous knockout cells. In contrast, *Nfe2l2, Sod1* and *Hmox1* downregulation did not occur in the homozygous knockout cells. These results show that the reduction in *Nfe2l2, Sod1,* and *Hmox1* expression during cell transplantation was also partly mediated by miR‐155 *in vivo*. However, deleting miR‐155 did not completely suppress ROS generation, although ROS levels in the homozygous knockout cells were lower than that in the wild‐type and heterozygous cells. This result is quite acceptable because ROS generation can be induced by various cascades, and our results suggest that the role of miR‐155 is not to induce ROS generation, but to suppress antioxidant systems.

Finally, we performed *hsa‐mir‐155‐5p* mimic transfection in human MSCs to assess the function of miR‐155 in human cells. Consistent with the results obtained in mouse cells, *hsa‐mir‐155‐5p* mimic suppressed the expression of *C/EBP*β*, NFE2L2, SOD1,* and *HMOX1*, thereby increasing ROS levels in the transfected cells. These results suggest that, at least, some parts of the redox signaling pathway are also targeted by miR‐155 in humans.

Considering that miR‐155 expression is activated by inflammation, the molecular roles of miR‐155 presented herein may partly explain the correlation between inflammation and ROS generation in aging and various diseases. Age‐related neurological disorders such as Alzheimer's disease (AD) and Parkinson's disease (PD) have long been associated with free radical‐induced oxidative stress, and these are driven by the nonhomeostatic production of ROS (Jiang *et al*., 2016). Importantly, miR‐155 upregulation was also reported in these diseases (Guedes *et al*., 2014; Thome *et al*., 2016). Thus, it is highly probable that miR‐155‐5p is also involved in ROS generation in these diseases. Now, miRNA‐targeting therapies are an area of intense interest in the medical and pharmaceutical fields, and actually, many nucleotide compounds are in preclinical and clinical development. Interestingly, it has been demonstrated that anti‐miR‐21 inhibits mitochondrial ROS generation in the kidney (Gomez *et al*., 2015). Thus, targeting or inhibiting miRNAs could be an option for managing ROS, and miR‐155 may be a promising therapeutic target for diseases related to inflammation and ROS, and may be important for cell transplantation therapies.

In conclusion, our findings indicate that miR‐155‐5p is upregulated in aged BM and MSCs, and describe a novel mechanism underlying the role of miR‐155‐5p in ROS generation by suppressing antioxidant genes through an upstream transcription factor, C/EBPβ. Elucidating the biological functions and molecular regulation of inflammation, miR‐155 expression, and ROS generation in stem cells is of critical importance in the future. Identification of miR‐155 target genes will be an essential step. Our study was based on information from *in silico* database and analysis with computational prediction tools. Therefore, noncanonical miRNA targeting may be overlooked. However, Sod1 has been reported as a target of miR‐155 noncanonical binding (Loeb *et al*., 2012) and Hmox1 is regulated by miR‐155 in mice (Zhang *et al*., 2015). Multiple regulation mechanisms are likely to exist in important biological systems such as deoxygenation, and combinations of these molecules enable the fine‐tuning of amount, timing, and duration. Further understanding of such mechanisms will provide further refinement of the model of inflammation‐related stresses and cellular robustness as well as information necessary for the development of novel therapeutic interventions for ROS‐related human diseases.

## Experimental procedures

### Antibodies and reagents

Antibodies and dilution conditions are presented in Table 1. Recombinant human IL1β (211‐11B; PeproTech, Rocky Hill, NJ, USA) and recombinant human TNFα (300‐01A; PeproTech) were prepared according to the manufacturer's instructions.

**Table 1:** Antibodies used in the present study

Antibody	Company	Application	Dilution
Pdgfrα (17‐1401‐81)	eBioscience	FACS	1:100
Sca1 (Clone : D7 , 11‐5981‐82)	eBioscience	FACS	1:100
CD44 (Clone : IM7 , 50‐0441‐U025)	TONBO Biosciences	FACS	1:100
CD105 (Clone : MJ7/18, 12‐1051‐81)	eBioscience	FACS	1:100
Isotype control, PE (550589)	BD Pharmingen™	FACS	
Isotype control, APC (17‐4321‐81)	eBioscience	FACS	
Isotype control, FITC (554001)	BD Pharmingen™	FACS	
NFE2L2 (sc‐722)	Santa Cruz Biotechnology	WB	1/1000 in Immuno‐enhancer
SOD1 (sc‐8637)	Santa Cruz Biotechnology	WB	1/1000 in Immuno‐enhancer
HMOX1 (sc‐136960)	Santa Cruz Biotechnology	WB	1/1000 in Immuno‐enhancer
IL1β (sc‐7834)	Santa Cruz Biotechnology	WB	1/1000 in Immuno‐enhancer
Tnfα (HP8001)	Hycult Biotech	WB	1/1000 in Immuno‐enhancer
GAPDH (sc‐25778)	Santa Cruz Biotechnology	WB	1/5000 in 0.2% Tween‐TBS containing 10% Block Ace
C/ebpβ (sc‐150x)	Santa Cruz Biotechnology	ChIP	2 μg
Normal mouse IgG (sc‐2025)	Santa Cruz Biotechnology	ChIP	2 μg

### Ethics statement

All procedures involving animals were approved by the Institutional Animal Care and Use Committee at Kindai University and were performed in accordance with institutional guidelines and regulations.

### Detection of ROS in the BM tissues of young and aged mice

Four‐week‐old (young) and 1.5‐year‐old (aged) C57BL/6N male mice were used for the experiment. BM tissues were prepared as previously reported (Zhu *et al*., 2010). Briefly, mice were euthanized and long bones were collected from the hindlimbs. Muscles, ligaments, and tendons were carefully removed using micro‐dissecting scissors. The long bones were carefully scrubbed to remove the residual soft tissues and transferred to a 100‐mm sterile culture dish with 10 mL α‐MEM on ice. The BM was flushed out of the long bones with α‐MEM twice. ROS levels were analyzed using CellROX Green Reagent (Thermo Fisher Scientific, Waltham, MA, USA) and FACS Canto^™^ II (BD Biosciences, Franklin Lakes, NJ, USA).

### Isolation of the MSC fraction from BM tissues

BM tissues were prepared as described above. The residual long bones were cut into small pieces around 2–3 mm^3^ and treated with collagenase type II for 15 min. Then, dissociated tissues were washed twice with PBS(‐) and reacted with anti‐PDGFRα (eBioscience, San Diego, CA, USA) and anti‐Sca1 (eBioscience) for FACS isolation of the MSCs. As a negative control, cells were reacted with PE‐ or FITC‐conjugated isotype IgGs (eBioscience). The double‐positive MSCs were sorted using FACS Aria II (BD Biosciences).

### Establishment and culture of mouse MSCs

BM tissues were collected from 6 weeks old C57BL/6N male mice (CLEA, Tokyo, Japan) as described above. Single‐cell suspensions containing MSCs were plated onto cell culture dishes (Sumilon, Sumitomo Bakelite Co. Ltd., Tokyo, Japan) and cultured in α‐MEM (Wako, Tokyo, Japan) supplemented with 200 mm L‐glutamine, 10% fetal bovine serum (Hyclone, Logan, UT, USA) under 5%CO_2_ and 5%O_2_ at 37°C. On day 2 of culture, the medium was replaced to remove dead cells and debris. After 10 days of culture, MSCs that formed colonies of fibroblastic cells were disaggregated by treatment with TrypLE Express (Thermo Fisher Scientific) and sorted by FACS using PDGFRα and Sca1 antibodies. Double‐positive cells were cultured in fresh medium. MSCs were passaged no more than twice for use in subsequent experiments.

### Real‐time RT–PCR

Total RNA was collected from MSCs using TRI Reagent^®^ (Molecular Research Center Inc., Cincinnati, OH, USA) and reverse‐transcribed with the PrimeScript^®^ RT Master Mix Kit (TAKARA Bio Inc., Shiga, Japan). Quantitative real‐time PCR of total cDNA was performed using Perfect real‐time SYBR green II (TAKARA). PCR amplifications were performed on a Thermal Cycler Dice^®^ Real Time System Single at 95°C for 20 s followed by 40 cycles at 95°C for 5 s and 60°C for 30 s. To quantify the relative expression of each gene, the Ct (threshold cycle) values were normalized to that of *Gapdh* and compared with a calibrator using the ΔΔCt method (ΔΔCt = ΔCt _sample_ − ΔCt _control_). To prevent amplification of contaminating genomic DNA, we designed all primers to span at least one intron. Data are expressed as mean values ± SD of six animals in *in vivo* experiments and three replicates in *in vitro* experiments. Statistical significance was evaluated by Student's t‐test with JMP software version 10.0.0 (SAS Institute, Cary, NC, USA). Primer sequences are listed in Table 2. For miRNA qPCR, total RNA prepared as above was reverse‐transcribed using the Universal cDNA synthesis Kit II (Exiqon, Inc., Vedbaek, Denmark). The resulting cDNA was diluted 1:50 for the qPCR. PCR was performed in a ExiLENT SYBR^®^ Green master mix (Exiqon) with the miRCURY LNA PCR primer sets (Exiqon): mmu‐miR‐15‐5p (ID 205930) and U6 snRNA (ID 203907).

**Table 2:** Primer sequences for quantitative RT–PCR, ChIP‐PCR, and genotyping in the present study

qRT–PCR		
Mouse	Forward	Reverse
*Il1*β	AGGATGAGGACATGAGCAC	ACGTCACACACCAGCAGGTTATC
*Tnf*α	ACGCTCTTCTGTCTACTGAACTTC	TGAGGGTCTGGGCCATAGAAC
*Il6*	AGGCTTAATTACACATGTTCTCTG	TCATCGTTGTTCATACAATCAG
*Nfe2l2*	AGACACCAGTGGATCCGCC	TACAAATGGGAATGTCTCTGC
*Sod1*	ATACTGATGGACGTGGAACC	AACCATCCACTTCGAGCAG
*Hmox1*	ACAGGGTGACAGAAGA	ACTCTGGTCTTTGTGT
*Bmi1*	TTTATGCAGCTCACCCGTC	CTCCTCATCTGCAACTTCTCC
*C/ebp*β	AAGAAGACGGTGGACAAGCTG	TGCTCCACCTTCTTCTGCAGC
*Gapdh*	TGGAGTCTACTGGTGTCTTC	TCTCGTGGTTCACACCCATC

Human	Forward	Reverse
*NFE2L2*	AGCACATCCAGTCAGAAACC	TCATCAAAGTACAAAGCATCTG
*SOD1*	TGGGCCAAAGGATGAA	TGATGCAATGGTCTCC
*HMOX1*	AGTTCATGAGGAACTTTCAG	TCTCCTTGTTGCGCTCAATCTC
*C/EBP*β	AGAAGGTGGAGCAGCTGTC	AGCTGCTTGAACAAGTTCC
*GAPDH*	AAGTATGACAACAGCCTCAAG	TCCACGATACCAAAGTTGTC

ChIP‐PCR		
Mouse	Forward	Reverse
*Nfe2l2 promoter*	ATTCTGCCTCAATTTTCTTAGC	ATCCAATTCCGACTCTTTTG
*Sod1 promoter*	ACTTTCACACTGCAACCG	TCGCTATTGTCTTCTAACTGTG
*Hmox1 promoter*	ACCAAGCAGAACTGTGAAC	AGAATTTGGGTCCTTTCC
*Positive control (Bmi1 promoter)*	ACTACACCGACACTAATTCCCAG	TCCAAAATGGCTCGGAGTCC
*Negative control (Patl2 intron)*	TCAGGTCCTAAGTCCTTTTCTGAGTGG	TGGACCCTTGGTGCCCTACTATCTAG

Genotyping	Forward	Reverse
*miR‐155 Knockout*	TCTGACATCTACGTTCATCC	ACTAACTGTGTGCGTACACAC

### Western blot (WB) analysis

MSCs from each experiment were homogenized in SDS buffer and centrifuged at 9,000 ×  *g* for 10 min at 4°C to remove debris. Aliquots were subjected to polyacrylamide gel electrophoresis followed by electrotransfer onto a PVDF membrane (Hybond‐P; GE Healthcare Japan, Tokyo, Japan). The blotted membranes were blocked overnight with Block Ace (Dainippon Sumitomo Pharma, Osaka, Japan) and then probed with primary antibodies overnight at 4°C. Detection was performed with horseradish peroxidase (HRP)‐conjugated secondary antibodies and Immunostar^®^ LD (Wako) detection reagents.

### *In silico* analysis for identification of the miR‐155 target genes

To identify the target of miR‐155, which can regulate the antioxidant genes, we performed target search using microT‐CDS v5.0 (http://diana.imis.athena-innovation.gr/DianaTools/index.php?r=microT_CDS/index), TargetScan (http://www.targetscan.org/vert_71/), and miRPath (https://mpd.bioinf.uni-sb.de). *In silico* ChIP‐seq analysis was performed using ChIP‐Atlas (DBCLS, Tokyo, Japan and Kyusyu University, Fukuoka, Japan) and Integrative Genomics Viewer Ver2.3.89 (Broad Institute, Cambridge, MA, USA) with GEO dataset GSE79813 (Meyer *et al*., 2016).

### Transfection of plasmids into the mouse MSCs

Prior to transfection, MSCs were dissociated using TrypLE Express, washed twice with PBS, and placed in 100 μL of Opti‐MEM (Thermo Fisher Scientific). MSCs were then transfected with miExpress™ EGFP‐mmu‐miR‐155 plasmid (GeneCopoeia Inc. Rockville, MD, USA) or pPBQM‐mmu‐miR‐155 plasmid (a gift from Dr. Martin Lotz) using a CUY21 electroporator (NEPA Gene, Tokyo, Japan). In the miExpress system, the precursor miRNA is placed in the 3′UTR region of the EGFP gene. After transfection, the precursors were co‐expressed with EGFP and processed in the cells. We used a scrambled control sequence expression plasmid (CmiR0001‐MR04; GeneCopoeia, Inc.) and a mmu‐miR‐155‐5p precursor expression plasmid (MmiR3427‐MR04, GeneCopoeia, Inc.). The pPBQM‐miR‐155 plasmid consists of the precursor sequence of mmu‐miR‐155 controlled by a cumate‐gene switch (Mullick *et al*., 2006), an EF1‐CymR repressor cassette, and a piggyback transposon backbone. The cumate‐gene switch was activated by adding 30 μg mL^−1^ cumate (QM100A‐1; System Bioscience Inc., Palo Alto, CA, USA). The C/ebpβ expression plasmid was from Addgene (#12557, Cambridge, MA, USA).

For mimic miRNA transfection experiments in human MSCs, we used a miRNA mimic for *hsa‐miR‐155‐5p* (*mir*Vana^®^ miRNA mimic, MC12601, Thermo Fisher Scientific) with Lipofectamine^®^ RNAiMAX (Thermo Fisher Scientific) following the manufacturer's instructions.

### Chromatin Immunoprecipitation (ChIP) assays

ChIP assays were performed using a ChIP‐IT Express Magnetic Chromatin Immunoprecipitation kit (53009; Active motif, Carlsbad, CA, USA) following the manufacturer's instructions. The chromatin was immunoprecipitated at 4°C overnight using 2 μg of anti‐rat C/ebpβ antibody (sc‐150x; Santa Cruz Biotechnology, Santa Cruz, CA, USA) and normal mouse IgG antibodies (Santa Cruz Biotechnology). The immunoprecipitates were collected using magnetic protein G beads and then washed three times. The immunoprecipitated DNA was used for ChIP‐PCR with primers in Table 2.

### Transplantation of MSCs

MSCs were collected from 8‐week‐old C57BL/6‐Tg (CAG‐EGFP) male mice (Japan SLC Inc., Shizuoka, Japan) and cultured as described above. Surgical procedures were performed on 8‐week‐old C57BL/6N male mice (CLEA Japan Inc., Tokyo, Japan). The recipient animals were anesthetized using 2% isoflurane. Following anesthesia, posterior biceps femoral (PBF) muscles were opened aseptically, a slit of 2 mm (width) × 3 mm (depth) was made using a scalpel. The mice were subsequently injected into the slit with 1 × 10^5^ MSCs from EGFP mice suspended in 2 μL of PBS. After 2 days, the mice were euthanized and the PBF muscles were collected for FACS assessment.

### CellROX analysis by FACS analysis and sorting of the GFP positive transplanted cells

To analyze ROS levels in transplanted cells, PBF muscles were minced with scalpels and incubated in α‐MEM containing collagenase and CellROX‐DeepRed (Thermo Fisher Scientific). After 30 min of incubation, the dissociated cells were collected, filtered through a 40‐μm cell strainer (Greiner Bio‐one, Frickenhausen, Germany), washed twice with PBS, and sorted by FACS aria. Transplanted cells were discriminated by GFP expression with a 530‐nm filter, and ROS signals (CellROX‐DeepRed) were analyzed with a 585‐nm filter. For gene expression analysis, GFP‐expressing cells were sorted and stored in liquid nitrogen. In this experiment, nontransplanted controls were prepared as follows: Cultured MSCs, which were collected from same mice with the transplanted cells, were dissociated by TrypLE Express, washed twice with PBS, mixed with minced PBF muscles collected from healthy mice, treated with collagenase containing CellROX‐DeepRed, filtered, washed, and analyzed with FACS at the same time as the transplanted cells.

### Generation of miR‐155 knockout ESCs and induction of differentiation into MSCs

To avoid side effects of the truncated RNA, we deleted the coding region of miR‐155 using two guide RNAs; guide 1, GTGGAACAAATGGCCACCGT and guide 2, GTTGCATATCCCTTATCCTC. To produce the knockout cell lines, we cultured a C57BL/6J ES cell line as previously reported (Teramura *et al*., 2010). For the CRISPR‐Cas9 treatment, the ESCs were dissociated with TrypLE Express, washed twice with Opti‐MEM, and resuspended in 100 μL of Opti‐MEM containing 7.5 μg of MLM3636 guide RNA expression plasmids and 5 μg Cas9 expression plasmid. MLM3636 plasmid (Addgene # 43860), and pSpCas9(BB)‐2A‐Puro (PX459) V2.0 (Addgene # 62988). After 1 μg μL^−1^ puromycin selection for 10 days, the resulting transgenic ESCs were cloned and genotyped. For genomic PCR for genotyping, ESCs were digested by KAPA Express Extract (Nippon Genetics Co, Ltd., Tokyo, Japan) diluted in Tris–EDTA. Genomic PCR was performed using KOD FX Neo DNA polymerase (TOYOBO Co. Ltd., Osaka, Japan) with genotyping primers in Table 2.

### Differentiation of MSCs from mouse ES cells

The wild‐type (WT), hetero, and homozygous knockout ESCs were induced to differentiate into MSCs *in vitro* as previously reported (Teramura *et al*., 2010). Briefly, dissociated ESCs were cultured in 60‐mm nonadherent culture dishes (Sumitomo Bakelite Co., Ltd.) for 4 days. Then, embryoid bodies obtained from the suspension culture were harvested on matrigel‐coated dishes and cultured in 10% FBS‐DMEM supplemented with 10^‐7^ m retinoic acid (SIGMA‐Aldrich, St Louis, MO, USA) in 5% O_2_ hypoxic condition for 10 days. After two passages in the MSC‐medium in hypoxic conditions, the induced MSCs were validated by FACS analysis using anti‐PDGFRα (eBioscience), anti‐CD44 (50‐0441‐U025; TONBO Biosciences, Kobe, Japan), and anti‐CD105 (12‐1051‐81, eBioscience) antibodies, confirmed MSC markers, and used for subsequent experiments.

### Culture of human MSCs

Human BM‐MSCs were purchased from Lonza (Walkersville, MD, USA) and cultured in 10% FBS α‐MEM under 5% CO_2_ and 5% O_2_ at 37°C.

### Statistical analysis

Significant differences were detected by Tukey–Kramer HSD test or Student's t‐test, as appropriate. *P* values <0.05 were considered significant.

## Author contributions

T.T. and K.F. were responsible for conceptualization. T.T. designed the experiments. Y.O., T.T., T.T., K.O., and T.M. performed the experiments. Y.O. and T.T. provided data analysis and interpretation. Y.O. and T.T. wrote the manuscript. Y.O., T.T., and T.T. contributed equally to this work. All authors contributed to and approved of the final manuscript.

## Competing interests

The authors declare no competing financial interests.

## Funding

No funding information provided.

## Supporting information


**Fig. S1** Western blot for the Il1β, Tnfα, Nfe2l2, Sod1, and Hmox1 expression in young and aged BMs.
**Fig. S2** Expression of mmu‐miR‐155‐5p after cumate induction.
**Fig. S3** Expression of mmu‐miR‐155‐5p in the knocked‐out cells.
