# Supplementary figures and images for: miR‐155 induces ROS generation through downregulation of antioxidation‐related genes in mesenchymal stem cells
Source: Aging Cell. 2017 Oct 2;16(6):1369–80. doi: 10.1111/acel.12680 (PMC5676067; doi:10.1111/acel.12680)

Supporting figure 1

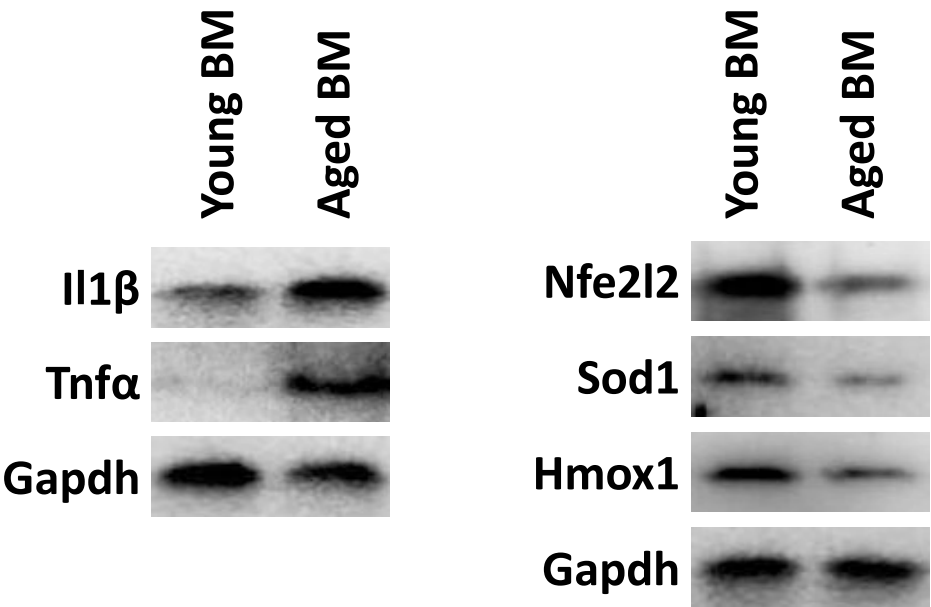

Supporting figure 2

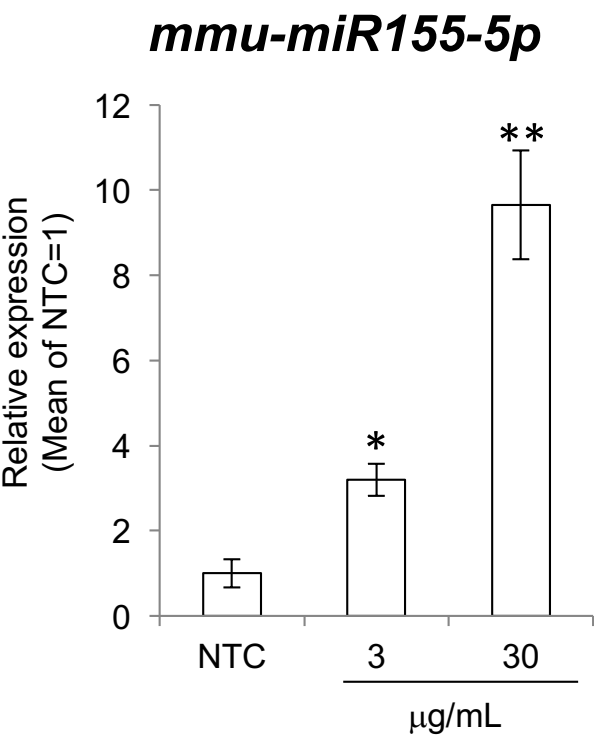

Supporting figure 3

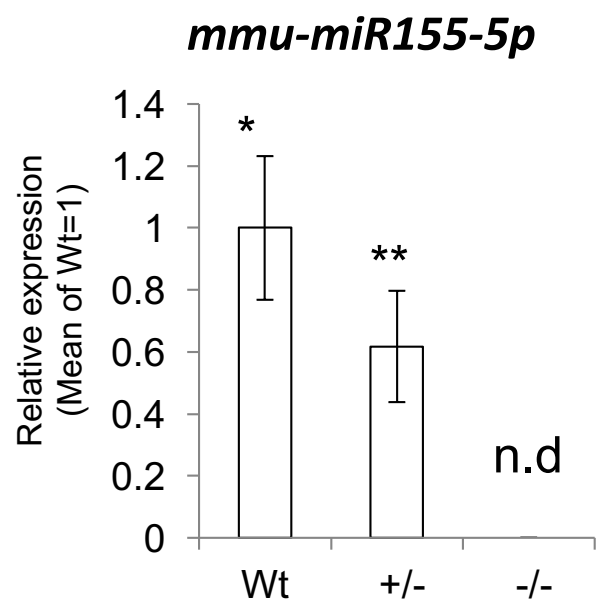

Supplement: Supplementary file 1 — Fig. S1 Western blot for the Il1β, Tnfα, Nfe2l2, Sod1, and Hmox1 expression in young and aged BMs. Fig. S2 Expression of mmu‐miR‐155‐5p after cumate induction. Fig. S3 Expression of mmu‐miR‐155‐5p in the knocked‐out cells. [file ACEL-16-1369-s001.pdf]
